# Electron Transfer Proteins as Electronic Conductors: Significance of the Metal and Its Binding Site in the Blue Cu Protein, Azurin

**DOI:** 10.1002/advs.201400026

**Published:** 2015-03-16

**Authors:** Nadav Amdursky, Lior Sepunaru, Sara Raichlin, Israel Pecht, Mordechai Sheves, David Cahen

**Affiliations:** ^1^ Departments of Materials and InterfacesWeizmann Institute of ScienceRehovot76100Israel; ^2^ Departments of Organic ChemistryWeizmann Institute of ScienceRehovot76100Israel; ^3^ Departments of ImmunologyWeizmann Institute of ScienceRehovot76100Israel

**Keywords:** compensation effect, electron transport, temperature dependence

## Abstract

Electron transfer (ET) proteins are biomolecules with specific functions, selected by evolution. As such they are attractive candidates for use in potential bioelectronic devices. The blue copper protein azurin (Az) is one of the most‐studied ET proteins. Traditional spectroscopic, electrochemical, and kinetic methods employed for studying ET to/from the protein's Cu ion have been complemented more recently by studies of electrical conduction through a monolayer of Az in the solid‐state, sandwiched between electrodes. As the latter type of measurement does not require involvement of a redox process, it also allows monitoring electronic transport (ETp) via redox‐inactive Az‐derivatives. Here, results of macroscopic ETp via redox‐active and ‐inactive Az derivatives, i.e., Cu(II) and Cu(I)‐Az, apo‐Az, Co(II)‐Az, Ni(II)‐Az, and Zn(II)‐Az are reported and compared. It is found that earlier reported temperature independence of ETp via Cu(II)‐Az (from 20 K until denaturation) is unique, as ETp via all other derivatives is thermally activated at temperatures >≈200 K. Conduction via Cu(I)‐Az shows unexpected temperature dependence >≈200 K, with currents decreasing at positive and increasing at negative bias. Taking all the data together we find a clear compensation effect of Az conduction around the Az denaturation temperature. This compensation can be understood by viewing the Az binding site as an electron trap, unless occupied by Cu(II), as in the native protein, with conduction of the native protein setting the upper transport efficiency limit.

## 1 Introduction

Electron transfer (ET) reactions are central to a wide range of biological processes, notably those of energy conversion such as the respiratory and photosynthetic chains. These employ mostly proteins, between which electrons are shuttled from one electron mediator to another by a redox process, driven by a free energy difference (driving force) between or within the mediators. The separation distance over which an electron is transferred within proteins can reach in some cases around 2.5 nm. One of the most intensively studied group of ET proteins is the blue copper proteins (e.g., plastocyanin, azurin, and the family of multi‐copper oxidases).^1^ The copper binding sites were shown to confer unique spectroscopic and thermodynamic properties on the copper ions.^2^ These properties attracted studies aiming at understanding their role in the ET via proteins.

Here we focus on the bacterial blue copper protein azurin (Az), functioning as an electron mediator in certain bacterial respiratory chains.^3^ Most of the studies of the ET process in Az were done in solution, using spectroscopy, mainly flash‐quench^4^ or pulse‐radiolysis^5^ techniques. A common denominator of these studies is monitoring the ET process between the Cu ion of Az and an intramolecular donor/acceptor. In another experimental approach for measuring intramolecular ET process in Az, the protein is bound to a conductive electrode and ET can be measured between the Cu ion and the electrode as a rate‐determining step of an electrochemical redox process in an electrochemical cell.^6^

In contrast, electronic transport (ETp) via the protein has more recently been studied by measuring conductance across Az, trapped between two electrodes in a solid‐state configuration.^7^ One pronounced difference between measuring ET in solution by spectroscopy or electrochemistry and measuring the ETp in a solid‐state configuration is that while the former requires a redox process to occur and, thus, the presence of a redox‐active center (such as the Cu ion, a disulphide bond or a bound external Ru complex), the latter does not.

A frequently used method of measuring solid‐state ETp is by nm‐scale probes, i.e., atomic force microscopy and scanning tunneling microscopy (AFM and STM, respectively).[[qv: 6a]],[[qv: 7a‐c,f‐h,k,l]] We have studied ETp via Az mainly by using a monolayer of the protein on an electronically conducting, ionically blocking substrate, and contacting the monolayer by macroscopic (≈500 μm in diameter) electrodes.[[qv: 7d,e,j]] Each of these approaches has its advantages and disadvantages: whereas the nm‐scale techniques allow the study of single/few Az molecules, the macroscopic technique allows characterization of the sample by a number of complementary methods, and the ETp measurement averages over a large (nominally ≈10^9^) ensemble of proteins. However, that measurement is also sensitive to defects in the monolayer. Because the contact area defines the current magnitude measured via the junction, the nm‐scale probe limits the currents that can be detected to orders of magnitude below those that can be measured via a macroscale electrode. Moreover, in AFM or STM measurements great care needs to be taken with respect to the force, and thus the pressure, that is applied to the protein, which may significantly affect the ETp mechanism via the protein.[[qv: 7h,k,l]],^8^ In both types of measurement care needs to be taken to limit the applied bias, so as to avoid changes in the sample by the applied electric field. Naturally this is easier to do in the macroscopic than in the nm‐scale method because of monitored larger signal and, thus, higher signal to noise ratio.

A striking early result of studying ETp via holo‐Az has been its temperature‐independence over the 20–360 K range.[[qv: 7e]] An important indication that Az retains its native structure under the particular conditions of the experiment is the irreversible drop in conduction, observed upon reaching the protein's denaturation temperature of ≈360 K. Further support emerged from results of the absorbance and fluorescence measurements.[[qv: 7j]] The temperature independence of ETp via Az is surprising because transport occurs over the protein's full length of ≈3.5 nm, between the electrodes, a distance well above an experimentally measurable current of electron tunneling via saturated organic molecules. It is at the limit of what has been reported for what is likely near‐resonance tunneling via conjugated systems,^9^ especially at room temperature (RT) or above. At these temperatures, a thermally activated process seems more likely as dominant transport mechanism. Furthermore, a 35 Å electron tunneling distance has never been observed before in proteins. The main concern that the ETp is via the Cu(II)‐Az and not via pin‐holes within the monolayer was validated by (a) nm‐scaled measurement using conductive‐probe (CP)–AFM which indicate similar ETp properties (both in current density magnitude and temperature independence),[[qv: 7h]] (b) ETp via the junctions without the protein was three orders of magnitude higher.[[qv: 7e,j]] This rather exceptional ETp process is further underscored by the observations that removal of the Cu ion from Az binding site,[[qv: 7e]] H to D isotopic exchange in the protein[[qv: 7d]] or even applying on it a relatively low external pressure by an AFM tip[[qv: 7h]] changes the temperature‐independent ETp process of holo‐Az to a thermally activated one at temperatures above ≈160 K.

As it is the metal ion (Cu) that assures the biological function of Az, imparting redox activity to the protein, we focus in this study on the role that the metal‐ion, its binding site, and oxidation state play in the ETp process via Az and how it affects the mechanism of ETp. The results are considered in perspective, based on our earlier studies, on other literature ETp data, as well as on the knowledge gained from the extensive ET studies.

## 2 Results and Discussion

### 2.1 The Cu Binding Site of Az and Its Role in ET

The Cu ion can be removed from holo‐Az to yield apo‐Az, which has a 3D structure that is very similar to that of holo‐Az.^50^ The metal binding site of Az has been shown to also bind other metal ions, such as Ni(II), Co(II), Zn(II), Hg(II), and Au(I).[[qv: 50b]],^10^ The binding site geometry of the Ni(II)‐, Co(II)‐, and Zn(II)‐ions was found to be similar to that of Cu(II)‐Az (no crystal structure is yet available for Hg(II)‐ or Au(I)‐Az).[[qv: 50b]],[[qv: 11a,d,e]],^11^ Though Ni(II) and Co(II) are redox‐active ions, both Ni‐Az and Co‐Az are redox‐inactive in aqueous solutions.[[qv: 12b]] Accordingly, ET measurements could be performed only with Cu‐Az.[[qv: 12b]] Because ETp measurement does not require a redox process, it provides the possibility to compare ETp via Cu‐Az to that via its derivatives with different metal ions, as well as via the likewise redox‐inactive apo‐Az.

### 2.2 The Role of the Metal Ion in the ETp Process

**Figure 1:**
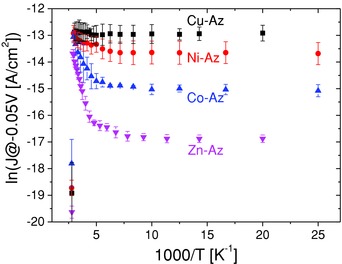
ln(*J*) versus 1000/*T* of Cu‐Az, Ni‐Az, Co‐Az, and Zn‐Az at −50 mV bias. The data points in the lower left corner represent the irreversible drop in current, which are ascribed to denaturation of the proteins. The top contacts were LOFO Au pads.

**Figure 2:**
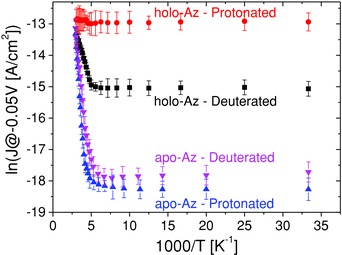
ln(*J*) versus 1000/*T* plots of holo‐Az, apo‐Az, and their deuterated forms at −50 mV bias. The top contacts were Hg drops.

**Figure 3:**
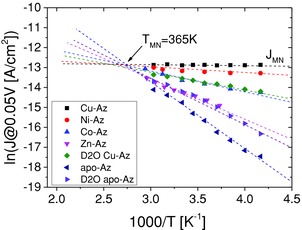
Summary of all temperature‐dependent current data, plotted as the natural logarithm of the current density [A cm^−2^] at −50 mV applied bias versus 1000/*T* [K^−1^]. It can be seen that the data fit the compensation or MNR. Cu(II)‐Az is included even though its ETp is not thermally activated. *T*_MN_ is the characteristic compensation temperature, found for Az, and is ≈365 K. *J*_MN_ is the (temperature‐independent) current density @ −50 mV through the Cu(II)‐Az junction. Data are from experiments with Au (LOFO) and Hg top contacts. The error bars for the various variants can be observed in Figures 1 and 2.

**Figure 4:**
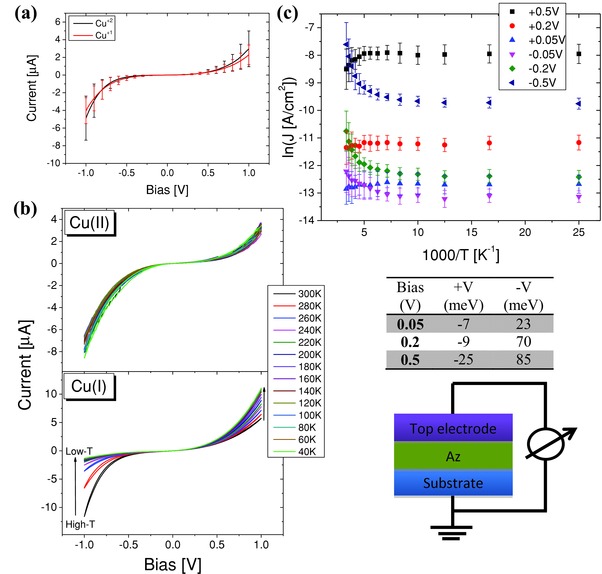
a) *I*–*V* characteristics of Cu(I) and Cu(II) Az junctions at RT. b) *I*–*V* for Cu(II) (top) and Cu(I) (bottom) Az junctions at several temperatures. c) ln(*J*) versus 1000/*T* plots for Cu(I) Az junctions at several indicated bias voltages. The table presents the activation energies of Cu(I) Az at the different measured biases (calculated by fitting to the Arrhenius equation). The scheme illustrates the electrical circuit of the molecular junction. The top contacts for the results in panel ) are Hg, b) Au (LOFO), and c) both Au (LOFO) and Hg.

Prior to the protein immobilization, current‐voltage (*I–V)* and temperature dependent *I–V* (*I–V–T*) were performed on junctions comprised of only the electrodes and the MPTMS linker (i.e., without a protein layer), as reported and discussed previously.[[qv: 7e,j]] ETp via such reference junctions (i.e., current density at a given bias voltage) was several orders of magnitude higher than for junctions with proteins. Linker‐only junctions showed temperature independent ETp, as expected from a relatively narrow tunneling barrier via a short three‐carbon chain and ≤1 nm Si oxide layer. Next, a series of *I–V* scans were recorded at several temperatures for each Az variant. Then, the current density (*J*) at ±50 mV (the lowest bias with acceptable signal/noise, and covering a 100 mV range over which the *I–V* dependence is essentially ohmic, i.e., linear) is presented as a function of inverse temperature (ln*J* – 1000/*T*, **Figures** **1**–**4**). If the process is thermally activated, its activation energy, *E*_a_, can be calculated by fitting the data from the thermally activated region to an Arrhenius equation. As can be seen by comparing the *J–T* characteristics of Cu‐, Co‐, Ni‐, and Zn‐Az (Figure 1), the bound metal ion plays a significant role in the ETp process. Most strikingly, while ETp via Cu‐Az is temperature‐independent, the ETp via Ni‐, Co‐, or Zn‐Az contains a thermally activated high temperature range with the respective transition temperatures of 180, 190, and 205 K, and with calculated *E*_a_ of 20, 70, and 150 meV, respectively (**Table** **1**).

**Table 1:** Solid‐state ETp via Az

Measuring system	Variant [force]		Activation energy [meV]	Transition to temp. dependence, *T*_c_ [K]	Current density @ *T* < *T*_c_ a)	Denaturation temperatureb) [K]
Macroscopic electrodes	Cu(II)[[qv: 7e]]		0	None	2.3 ± 0.4 × 10^−6^	360
	Deuterated Cu(II)[[qv: 7d]]		80 ± 18	180	2.7 ± 0.3 × 10^−7^	–
	Cu(I)		21 ± 9c)	200	2.0 ± 0.8 × 10^−6^ c)	–
	Ni(II)		20 ± 12	180	1.0 ± 0.3 × 10^−6^	360
	Co(II)		70 ± 7	190	3.1 ± 0.6 × 10^−7^	360
	Zn(II)		150 ± 22	205	5.1 ± 1.0 × 10^−8^	358
	Metal‐free (apo)[[qv: 7e]]		320 ± 31	205	2.8 ± 0.8 × 10^−8^	358
	Deuterated metal‐free		220 ± 37	195	4.1 ± 1.3 × 10^−8^	–
CP–AFM[[qv: 7h]]	Cu(II)	[6 nN]	0	None	5.8 ± 2.6 × 10^−10^	372
		[9 nN]	1010 ± 180	310	1.6 ± 0.6 × 10^−9^	–
		[12 nN]	1080 ± 205	310	2.7 ± 1.1 × 10^−9^	–
		[6 nN]	520 ± 115	–d)	1.4 ± 0.7 × 10^−11^ e)	–
	Metal‐free	[9 nN]	490 ± 120	−d)	6.2 ± 3.4 × 10^−11^ e)	368
		[12 nN]	500 ± 105	−d)	3.9 ± 1.9 × 10^−10^ e)	–

^a)^The current densities, measured with the macroscopic electrodes, are those at −0.05 V; the units are A cm^−2^. CP–AFM current densities are in A nm^−2^ (a detailed explanation for the determination of the AFM tip surface area can be found in ref. [[qv: 7h]] and were measured at +0.5 V because of the poor S/N at lower bias voltages in measurements with nanoscopic contact area;

^b)^We give here the temperatures where, upon heating, the current drops irreversibly. The thermal denaturation temperature of Cu(II)Az in solution is around 355 K and those of the other derivatives range between 355 and 365 K.^12^ We ascribe the higher temperatures seen in the nanoscopic (CP–AFM) than in the macroscopic experiments to differences between the two setups, where uniform temperatures are much harder to achieve in the SPM than in the probe station;

^c)^We present the values for Cu(I), measured at −0.05 V, but as discussed later, and unlike the case for the other Az derivatives, the values for Cu(I) differ, consistently, between negative and positive bias;

^d)^The temperature range of the CP–AFM measurements is 250–370 K, and, therefore, we could not decrease the temperature enough to reach *T*_c_ for apo‐Az;

^e)^These are values at 278 K because as this was the lowest temperature at which the CP–AFM data were (and could be) measured.

Thermally activated ETp at high temperatures and temperature‐independent ETp at low temperatures can be expected for transport across >2.5 nm of a relatively flexible system such as a protein, as for other molecular junctions.^13^ Proteins have numerous vibrational modes that are excited at higher temperatures, which can induce vibration‐assisted ETp, also known as incoherent hopping.^14^ As temperature is lowered, these vibration modes freeze out in what is known as a transition to the glassy state.^15^ This transition may be coupled to that of thermally activated to temperature‐independent conduction at low temperatures and can be described as off‐resonance tunneling via a molecular barrier. Indeed, we found this type of behavior for all of the proteins measured so far, including native and modified CytC, bacteriorhodopsin, bovine‐ and human‐serum albumins, with one notable exception, namely holo‐Az,[[qv: 7d,e]],^16^

The findings that ETp characteristics change considerably upon replacing the native Cu(II) ion of Az by Co(II), Ni(II), or Zn(II) imply that the nature of the metal ion is crucial for ETp via Az. It seems that, even though the coordination geometry of Cu‐, Ni‐, Co‐, and Zn‐Az are similar (as evident from the X‐ray crystal structures), the distinct chemical nature of the metal ions, which likely will affect the electronic structure of the bound metal and its coordination shell, is the main cause for the observed differences in the ETp. The coordination of the binding site of Az has also an important role in the ability of Az to mediate ET in spectroscopic and electrochemical experiments in solution. However, as stated above, Cu(II)‐Az is the only Az derivative that is known to be redox‐active,[[qv: 12b]] and all ET studies of Az in aqueous solutions were conducted with this derivative. Studies of single‐site mutations of Az have shown that relatively small structural changes in the first and second coordination sphere of the Cu ion cause marked changes in the protein's ET characteristics, as shown by electrochemical^17^ and by pulse radiolysis studies.^18^ Most probably, the ETp differences observed in the high temperature regime (T ≈ >200 K) also reflect metal‐induced structural changes in the protein's vibrational modes.^19^ The result for Cu(II)‐Az differ from those of all other variants by the comparatively higher current magnitude and the insensitivity of the ETp efficiency to temperature (Figure 1, black squares). The ETp mechanism that fits this observation is tunneling which is near to resonance (nearer than the other Az variants). This is further supported by previous work of Venkatramani et al.,^20^ which showed that efficient ETp and ET is correlated with the increase of guanine content of peptide nucleic acid, PNA. This was ascribed to near‐resonance tunneling in the PNA, both in electrochemical and solid state measurement configurations. In a similar fashion, in our case, the presence of Cu(II) may support an electronic state which is energetically close to the Fermi level of the electrodes (for a schematic 1D energy diagram see Figure S3, Supporting Information). The temperature independence of this process is further supported by results from a theoretical investigation by Segal et al.,^21^ who showed that in the case of small barrier between the electrode and the bridge (in this case due to the presence of Cu(II) ion), an activationless process should be seen.

The differences in ETp between the derivatives become more apparent at low temperature, where we observe nearly two orders of magnitude difference in current between Cu(II)‐Az and Zn‐Az (comparing the currents at *T* < *T*_c_; see Table 1). As stated above, we attribute the currents in the low‐temperature regime to off‐resonant tunneling via the protein. Thus, the different current magnitudes of the different Az variants represent different tunneling barriers of the molecular junction (see also Section 3.7).

ETp across apo‐Az was found to be similar to that across Zn‐Az, with identical *T*_C_, but with a higher thermal activation energy at *T* >*T*_C_ and lower tunneling currents at *T* < *T*_C_ (Figure 2 and Table 1). Indeed, apo‐Az exhibits the lowest current density < *T*_C_ and the highest activation energy >*T*_C_ of all studied Az derivatives, showing that even redox‐inactive metal ions that replace Cu facilitate ETp better than apo‐Az at almost all temperatures.

We note that all measurements reflect ETp via individual proteins and assume no (or negligible) intermolecular or cooperative effect. This is supported by the following:

- a) The temperature dependent ETp measurements for macro‐ and nanoscale agree with each other qualitatively.[[qv: 7h]]
- b) ETp via single Az reveal similar ETp efficiencies to the CP–AFM measurements.[[qv: 7a,l]]
- c) Clear qualitative differences in temperature dependence of ETp between nano‐(10s to 100s of molecules) junctions and single molecule junctions composed of identical conjugated molecules were found by Selzer et al.^22^ There, temperature dependence is observed only via single molecular junction, as the ensemble shows temperature‐independent transport. However, as noted above, for Az we do not find such differences.

### 2.3 Kinetic Isotope Effect (KIE) of ETp via Holo‐Az and Apo‐Az

Aiming at resolving a possible role of structural differences in the ETp, mainly in terms of hydrogen bonding and its effect on the protein conformation, we measured the H/D KIE of this process. The KIE on ET reactions is expressed in the Marcus' theory mainly by solvent reorganization energy, due to H/D exchange‐caused differences in the optical dielectric constant and refractive index.^23^ These differences between H_2_O and D_2_O are small and only a minor inverse KIE value (≈0.9) is expected.^24^ As our measurements were performed in the solid‐state and at reduced pressure (≈10^−3^ mbar), effects of the solvent will be limited to the protein's tightly bound water molecules, i.e., different from the case of a solvated protein. Thus, we can expect that the main or only contributions to the KIE are intraprotein ones of either high‐frequency modes (i.e., stretching or rocking of H/D‐bonds) or low‐frequency structural modes of the protein. Theoretically, these intramolecular contributions should yield a small positive KIE value (<1.2).^24^, ^25^

The intramolecular ET in azurin (in aqueous solution) between the Cu(II) site and the RSSR^−^ radical produced by pulse radiolysis was found to have a negative KIE of 0.7 (at RT).^18^ This inverse KIE value was tentatively rationalized by differences in the thermal expansion of Az between H_2_O and D_2_O. We found in our solid‐state ETp measurements a marked positive KIE value (2.4 at RT).[[qv: 7d]] Furthermore, unlike normal, nondeuterated holo‐Az, for which ETp is temperature independent, upon deuteration ETp via holo‐Az becomes thermally activated at >*T*_C_ = 180 K (Figure 2). This yields a temperature‐dependent KIE value that increases with decreasing temperature from 1.8 at 340 K to 9.1 <180 K. We attributed this large KIE value mainly to the isotope effect on low‐frequency structural modes.[[qv: 7d]]

To complement these results of KIE measurements of ETp via holo‐Az, similar measurements were conducted on ETp via apo‐Az (Figure 2). In contrast to holo‐Az, where a marked positive KIE was observed, the KIE of apo‐Az is small, ranging from 0.8 (at 340 K) to 0.6 (<130 K), i.e., similar to what was reported for ET in Az in aqueous solution.^18^

In essence, the observed large positive KIE can be ascribed to the exceptional temperature independence of ETp via holo‐Az. Once this intrinsic cause for the temperature independence, which is assigned to the Cu(II) ion (see below), is eliminated, the KIE has much smaller values. Its value is probably due to an effect (direct or indirect) of H‐bonding and/or H‐involving interactions on the ETp.

We have previously suggested that the high KIE value of the ETp via holo‐Az is due to a change in the flexibility of the protein. We hypothesized that the deuterated holo‐Az has a greater degree of flexibility, possessing lower energy frequency modes, ℏω, than the more rigid, (protium) holo‐Az, which will result in a thermally activated ETp, if $\hbar \omega \leq 2k_{B}T$.^26^ It was shown by NMR^27^ that apo‐Az is much more flexible than holo‐Az, especially around the metal's first coordination sphere. Thus, it may well be that for a more flexible protein, such as apo‐Az, the KIE on the ETp process will be much smaller than for a more rigid protein as holo‐Az.

In addition, and as we show in this study, the coupling of the metal ion to the electrode plays an important role in the efficiency of the ETp and affects its temperature dependence. Thus, while even as minor a change in holo‐Az as deuteration induces a large KIE of the ETp, the KIE in apo‐Az is very small and resembles the one that was reported in solution.^18^

### 2.4 Compensation Effect in ETp via Az

From a plot of the temperature‐dependent ETp data for all Az variants that we studied as ln(*J*) versus 1000/*T* (Figure 3) we see that ETp across the Az system obeys a general relation, in the case of electronic conduction also called the Meyer–Neldel rule (MNR). This rule, first reported in 1938, was based on analyses of electrical transport measurements of oxide semiconductors,^28^ where it was observed that the activation energy (*E*_a_) of the conduction is proportional to the temperature‐independent preexponential factor. This means that if the conduction is thermally activated: $I=I_{0}\exp(-E_{a}/k_{B}T)$, the prefactor $I_{0}\propto \exp(E_{a}/k_{B}T_{\mathrm{MN}})$, where *T*_MN_ is the Meyer–Neldel temperature.^29^ Due to this proportionality, the MNR is frequently referred to as the compensation rule, where the thermal activation energy for conductivity and the temperature‐independent prefactor compensate each other. Among the models that have been proposed for the origin of this behavior (apart from doubting its existence) we note the presence of trap states in the examined conductors,^30^ which consequently can be also related to (thermally activated) hopping (small polaron conduction) that involve these states.^29^, ^31^ Rosenberg and colleagues found that ETp across hemoglobin crystals in different hydration states follow the compensation rule.^32^

Our results of the ETp via the different Az‐derivatives (Figure 3) in the thermally activated regime show clear compensation behavior with a characteristic temperature around 365 K and a compensation current (*J*_MN_) equal to the temperature‐independent one of Cu(II)‐Az. Such behavior is consistent with a thermally activated solid‐state ETp mechanism with hopping via traps, where, in the case of Az, the trap may be identified as the metal binding site, if it is not occupied by Cu(II). As the results of the apo‐Az variants also follow the MNR, not only replacing the Cu(II) but also its removal generates a trap state in the protein molecular junction, i.e., we can ascribe such trap to the absence of the (functionally) “correct” metal ion, viz., Cu(II).

Kemeny and Rosenberg analyzed results of organic semiconductors, using a small polaron conduction model to arrive at a relation between T_MN_ and the Debye temperature.^32^, ^33^ Dyre,^34^ using a more general model of a disordered system with an exponential probability distribution of energy barriers suggested that *T*_MN_ constitutes the temperature of a structural change, a type of “glass transition temperature.” In our case, we view *T*_c_, the temperature at which ETp changes between thermally activated and temperature‐independent (except for Cu(II)‐Az) as a glass‐transition – like one, where the internal vibrations are frozen out. *T*_MN_ is then best viewed as the temperature where the system undergoes another structural change (see below). Conduction in a disordered medium is also at the heart of a model that was used to explain MNR, as found in recent experiments on organic semiconductors.^35^

In light of those early studies and the recent model it is remarkable that *T*_MN_ of Az is close to the protein's denaturation temperatures, which may be related to the thermal stability of the metal binding site. This finding is even more remarkable in light of other work of Rosenberg et al., where they reviewed the then existing literature and noted that also the correlation of thermal cell death with protein denaturation,^36^ as well as thermal denaturation of a series of visual pigments and proteins,^37^ if viewed as activated processes, follow the compensation rule. The former^36^ was attacked as artifact,^38^ but in the ensuing polemic, defended by the authors and others.^39^ Most interesting is the (nonreferenced) concluding remark in the 1971 paper of Rosenberg,^37^ drawing attention to the correspondence between compensation in electrical conduction and denaturation temperatures for (unspecified) proteins.^40^

Recently, theoretical investigations showed that a compensation effect exists for ET in the cytochrome c peroxidase:cytochrome C complex.^41^ Their analysis, which relies on results from ET experiments, suggests that with decreasing electronic coupling (due to increasing distance between donor and acceptor) there is an increase in the reorganization energy. This eventually leads to similar ET rates at RT (the ET process in question occurred in the inverted Marcus regime). This proposed compensation in the ET is qualitatively similar to the quantitative compensation effect that we find, which, in solid‐state conductors is also termed the Meyer–Neldel effect.

### 2.5 The Role of the Cu Oxidation State in the ETp Process

In the ET process to/from the Cu ion in Az, whether monitored by spectroscopy or by electrochemistry, the Cu ion undergoes a change in its oxidation state (Cu(II)+e −⇄Cu(I)). As this is not a requirement for solid state conduction, we compared the ETp via the oxidized form of Az (Cu(II)) to that via the reduced form (Cu(I)). Although the *I–V* curves at RT are very similar (Figure 4a), the temperature dependences of the measured *I–Vs* are strikingly different (Figure 4b). While Cu(II)‐Az exhibits temperature‐independent ETp, irrespective of applied bias (as shown in Figure 4b, top panel), the reduced form exhibits different, and quite unexpected temperature dependence (Figure 4b, bottom panel, and 4c). Whereas at applied negative bias (to the top contact) the currents are thermally activated >140 K, at positive bias we find inverse thermal dependences, i.e., currents decrease with increasing temperature. We are not aware of anything resembling this type of effect of bias polarity on the temperature dependence of currents in molecular bioelectronics. We note that we have previously observed an apparent negative activation energy of ETp via simple linear alkyl chains,^42^ which was ascribed mostly to a decrease of defects in the monolayer upon cooling. In that case no dependence on the polarity of the applied bias was observed. Apparent negative activation energy coupled to bias polarity was reported previously.^43^ The phenomenon was mostly related to temperature‐dependent conformational changes within the molecular junction, which may be the reason for this unique ETp via Cu(I)‐Az.

As can be seen from the 3D structural data (for an excellent compilation and general review of Az variants see ref. [[qv: 12b]]), the copper binding site in Az represents a structural intermediate between those of Cu(II) and Cu(I). Thus, applying a positive bias through the Hg or Au top electrode (see schematic in Figure 4), which is the one closest to the metal site, can lead to an “oxidative pull” on the Cu(I), i.e., polarization is applied to the contact closest to the copper ion. This electrostatic effect might affect the binding site, so as to bring it, in terms of electronic configuration, closer to the optimal Cu(II) site coordination. Therefore, at applied positive bias a near temperature independent transport via Cu(I) can be seen similarly to Cu(II) (see Figure 4c, +V). If the bias is negative on the top contact, it will be equivalent to a “reductive push” on the Cu(I) site, i.e., change it electrostatically away from the structure of Cu(II)‐Az. Furthermore, or alternatively, possibly when negative bias is applied the dominant charge carrier for ETp via Cu(I)‐Az may be electron rather than hole transport, at the higher temperatures (See Figure 4c, −V). The cause for such a switch may be broadening of the Cu(I) state and/or Fermi level broadening of the electrode's density of states at increasing temperatures.

### 2.6 External Pressure versus Internal “Strain”

Possible support for the notion that causing strain in the protein (by applying pressure) may cause a particular ETp behavior comes from studies where external pressure has been applied to the examined proteins: We have studied this by measuring the ETp dependence on both temperature and (tip‐induced) pressure, using CP–AFM.[[qv: 7h]] At low tip force (6 nN), the temperature dependence was similar to that observed, using macroscopic electrodes,[[qv: 7e]] namely, temperature‐independent ETp via holo‐Cu(II)‐Az, and thermally activated ETp via apo‐Az (Figure S4b, Supporting Information and Table 1). Intuitively, one expects that with increasing AFM tip pressure on the protein, the tip‐substrate separation distance will decreases, causing the current magnitude to increase, as is indeed observed for apo‐Az (Figure S4a, Supporting Information). As apo‐Az is more flexible than holo‐Az, the force effect on the measured current density is expected to be more pronounced in apo‐Az than in holo‐Az.

Upon increasing the tip force to 9 and 12 nN the thermally activated process via apo‐Az does not change, except for an increase in currents, which is explained by the decrease in the electrodes' separation distance. However, a tip force > 6 nN changed the holo‐Az ETp at *T* > 310 K from a temperature‐independent to thermally activated. This may be explained by assuming that the force, applied by the AFM tip, affects the metal binding site's coordination sphere. Indeed, it was shown theoretically^44^ and experimentally^27^ that changes in the Az binding site loop region, which is the relatively flexible part of the protein, have a major impact on the reorganization energy of the Cu binding site. The increased protein flexibility may allow better electrode–metal coupling and electronic overlap, which will lead to increased current. It was previously observed by us that ETp efficiency is significantly affected by the coupling of protein‐bound cofactors to the electrodes.^45^

Substituting Cu(II) in Az by other ions or removing it altogether, alters the first coordination sphere of the metal ion,[[qv: 12b]] and the metal‐ligand distances. Hence, the internal “strain” can be viewed in terms of a change in the coordination of the metal binding site. It will also have a lesser effect on the bond distances and angles in the second coordination sphere. This may cause some internal strain in the rest of the protein. While it is attractive to consider differences in ionic radii of Cu(II) and of the ions that substitute it, the experimentally determined bond lengths do not correlate with these radii. Using the Shannon effective oxide/fluoride and sulfide ionic radii,^46^ we find the following order: Ni(II) < Cu(II) ≈ Co(II) (using the high spin value for Co)< Zn(II) < Cu(I). Considering the rather unique coordination site of Az, it's not surprising that this order does not match the observed bond length changes, which show the largest averaged increase for Co(II), followed by Ni(II), while for Zn(II) and especially Cu(I) similar values to those for Cu(II)‐Az are found. Still, each substitution causes a significant change from that assumed to be the optimal coordination geometry.

### 2.7 Does a Redox Cycle Take Place as Part of the ETp Process?

While electronic charge carrier transport via hopping in a solid‐state conductor need not to entail a change in oxidation state, such a change is possible in rather insulating materials. It implies electron localization, i.e., electron residence on the hopping site for sufficiently long time to cause the full change in nuclear polarization that accompanies a change in oxidation state (e.g., from Cu(II) to Cu(I)). Such a situation can be described by small polaron hopping. If, instead, there is significant delocalization of the electronic charge, no redox process will occur and the situation can be described as large polaron hopping. Also if only electronic polarization with or without partial nuclear relaxation accompanies the charge movement, no (complete) redox process will occur. The mechanism will be different from tunneling, but if delocalization is large, some degree of band conduction can be invoked.

Results of our studies of ETp via Cyt C and Fe‐free CytC,[[qv: 17c]] as well as those via human serum albumin, doped with hemin and with Fe‐free hemin,[[qv: 17a,b]] taken together with the fundamentally different *J–T* behavior presented here for Cu(II)‐ and (I)‐Az, rule out the involvement of a complete redox process. Among the reasons for this conclusion, apart from the time‐scale of the process, is also the absence of mobile countercharges outside the protein (as in an electrochemical or spectroscopic experiment) that is required for electrostatic charge balance in the redox process. This is not to say that it is impossible to have a redox process in ETp, and indeed there are some reports of experiments, as well as theory on this.^47^

In the case of hopping with short residence time on a site, or off‐resonance tunneling via the protein, the energy difference (ΔE) between the lead's energy level and the virtual (or actual) bridge state will affect the conductance magnitude. Interestingly, the M(II) ←→ M(0) reduction potentials of Cu(II), Ni(II), Co(II), and Zn(II) correlate with the temperature‐independent currents at *T* < *T*_c_. The current densities are 2.3 × 10^−6^, 1.0 × 10^−6^, 3.4 × 10^−7^, and 5.1 × 10^−8^ A cm^−2^ and the M(II) ←→ M(0) reduction potentials are +0.34, −0.25, −0.28, and −0.76 V versus standard hydrogen electrode, for Cu, Ni, Co, and Zn, respectively.^48^ This leads us to suggest a correlation between the latter metal redox potentials and the HOMO (highest occupied molecular orbital) (or LUMO [lowest unoccupied molecular orbital]) states of the whole protein. In other words, the barrier height for ETp via Az may be related to the protein's redox potential, which will be strongly influenced by the presence of a redox‐active metal ion. This in turn can influence the conductance magnitude.

Similarly, the ETp activation energies via different Az variants correlate with the tunneling currents at *T* < *T*_C_ (which reflect the tunneling barrier height). As can be seen in Table 1 (and Figures 2 and 3), the higher is the activation energy, the lower are the tunneling currents at *T* < *T*_C_. These results favor the assumption that the transport is assisted by superexchange‐mediated tunneling at cryogenic temperatures and supports real residence of charge on a single hopping site at *T* > *T*_C_, which is the metal ion. The activation energies extracted (see Table 1) are ≤1 eV. This rather low barrier (compared to the commonly reported 2–3 eV) can be explained by (a) the presence of electronic coupling with the continuum of states in the contacting electrode, which lowers the energetic gap (between the electrode and the ion state) or (b) due to partial (and not full) vibrational relaxation.

The above explanations do not fit with the apparent negative activation energies observed at positive biases for Cu(I). These cannot be explained by a thermally activated single‐step process via the metal ion and other factors, as discussed in the previous section, may start to play a role.

## 3 Conclusions

The role of the metal binding site of Az in the ETp process has been investigated by solid‐state measurements using several derivatives of the protein. These included: 1) removing the Cu ion (apo‐Az); 2) replacing the Cu by Ni, Co, or Zn; 3) reducing the Cu(II) ion to the monovalent state. In addition, we studied the variants obtained by deuterating holo‐ and apo‐Az, and measuring the KIE of the ETp across each. Furthermore, we studied the effect on the ETp of applying an external pressure on the protein. All of these are possible in ETp measurements, which are, thus, quite different from ET measurements, which always involve the redox process of the Cu ion within Az.

All the above modifications affected the ETp via Az in different ways. The most pronounced change was the ETp transition from totally temperature independent (from 360 to 20 K), to thermally activated at *T* > 180–205 K, depending on the Az variant. The Az modification affects mostly the (super‐exchange‐mediated) tunneling process, which becomes less effective than for holo‐Az, i.e., at low temperatures the tunneling current through Cu(II)‐Az is higher than tunneling currents through the other Az variants. It is conceivable that the metal–electrode coupling is the dominant factor for transport and that at low temperatures the protein–(top)electrode coupling is weakened in the other Az variants, thereby leading to less effective ETp. The observation that deuteration also affected the tunneling process, indicates that the protein hydrogen bonding network affects the metal–electrode coupling and maintains specific protein conformation at low temperatures that allows for effective coupling in Cu(II)‐Az.

Combining all of present results of temperature‐dependent electronic conduction measurements across the different Az derivatives a clear compensation effect (apparent Meyer–Neldel behavior) is observed. The value of the compensation *current* equals that obtained for Cu(II)‐Az, i.e., the temperature‐independent current that flows across the holo‐Az. The compensation *temperature* that we find (by extrapolation) is around the denaturation temperature of all Az derivatives. This compensation behavior can be understood by viewing the Az binding site as a trap, except if it is occupied by Cu(II). We can speculate that if the systems would have been able to conduct up to the denaturation temperature then their conduction would have reached that of the natural protein, apparently the maximum ETp efficiency of the system.

We have further observed that the ETp via Cu(I)‐Az exhibits a rather unique and puzzling behavior where at positive biases the current decreases as the temperature increases (>200 K). We rationalize these findings in terms of possible structural strain within the metal binding site induced by the different experimental modifications or treatments of the protein, from possible changes in bond distances, different ion radii dimensions, electrostatic polarization by the biased electrodes, and the pressure exerted by an AFM tip.

Our results of the thermally activated ETp regime seem to exclude that a full redox change (as in ET reactions) accompanies electron hopping in our solid‐state ETp measurements. Rather, a vibration‐assisted hopping mechanism that does not involve redox (i.e., short resident time of the electron on the hopping site) is the most likely ETp mechanism at higher temperatures (*T* >*T*_C_). At low temperatures, upon the transition of the protein to its glassy‐state, the most‐likely operating mechanism is an off‐resonance super‐exchange type of tunneling.

## 4 Experimental Section

*Expression and Purification*: The plasmid for *Pseudomonas aeruginosa* Az was generously provided by Dr. Yuling Sheng from Caltech, CA, USA. After transformation of the plasmid into BL21(DE3), a single colony was picked from a lysogeny broth (LB)‐Agar + Amp plate and used to inoculate 2 × 4 mL terrific broth (TB) starter cultures containing 100 μg mL^−1^ ampicillin. The cultures were grown at 37 °C for ≈4–5 h (until they become slightly translucent) followed by sedimentation and resuspension in 6 mL TB. For overexpression, 8 mL of the resuspended starter culture was added to 8 L of TB, inoculated with 100 μg mL^−1^ of amp, and placed at 32 °C for O/N with vigorous shaking. Later, 1 × 10^−3^ m of isopropyl‐beta‐D‐thiogalactopyranoside (IPTG) was added to the culture, the temperature was raised to 37 °C, and the overexpression was proceed for five additional hours. For harvesting the cells, the cell pellets were resuspended in 2 × 200 mL of Tris/Sucrose/EDTA solution for 30 min. The cell pellets were spun down at 5000 RPM (rate‐per‐minute) for 20 min. The supernatant was removed and 50 mL of ice‐cold 500 × 10^−6^ m MgCl_2_ was added to each of the pellets and was stirred at 0 °C for ≈30 min. The ionic strength was restored by adding MgCl_2_ to a final concentration of ≈200 × 10^−3^ m and the cells remaining were sedimented at 12 000 RPM at 0 °C for 30 min. The supernatant was transferred into ≈200 mL of 500 × 10^−3^ m ammonium acetate pH 4.5 and was stirred for 20 min. A 100 × 10^−3^ m of CuSO_4_ was added to the ammonium acetate solution, where an intense blue color started to appear. The solution was spin at 12 000 RPM to remove any precipitation and the ionic strength was dropped to ≈25 × 10^−3^ m ammonium acetate to improve column binding. For final purification, the solution was loaded onto a chromatography column that was preequilibrated with 25 × 10^−3^ m ammonium acetate. The purified Az protein was loaded on an sodium dodecyl sulfate polyacrylamide gel electrophoresis (SDS–PAGE) gel (Figure S1, Supporting Information) to validate the purification and the molecular weight of the protein (≈14 KDa). The final Az solution was adjusted to a concentration of ≈1 mg mL^−1^.

*Apo‐Az and Ni‐, Co‐, Zn‐Az Preparation*: For preparation of apo‐Az, the above holo‐Az solution was dialyzed against 100 × 10−3 m KCN in 50 × 10−3 m ammonium acetate buffer, pH 8, for ≈5 h at RT. The dialysis bag was then moved for continuing dialysis against fresh 50 × 10−3 m ammonium acetate buffer, pH 8, for at least 24 h at 4 °C in order to remove the cyanide and then against 50 × 10−3 m ammonium acetate buffer, pH 4.6, for an additional 24 h at 4 °C.

For preparation of Ni‐, Co‐, and Zn‐Az, a solution of apo‐Az, prepared as described above, was placed into 50 × 10^−3^ m ammonium acetate buffer, pH 8, containing the sulfate hydrate salts of either Ni(II), Co(II), or Zn(II) that were added in a 10:1 (metal:protein) excess for at least 24 h of incubation at 4 °C. The excess of metal ions was removed by dialyzing the solution against 50 × 10^−3^ m ammonium acetate buffer, pH 4.6, for 24 h at 4 °C. The final concentration of the modified protein (≈0.7–1 mg mL^−1^), the removal of the Cu ion, and its replacement by the desired metal ion were determined by UV–vis spectra (Figure S2, Supporting Information, acquired by a Nanodrop 1000 system, Thermo scientific), the results of which are in line with previous works.^49^

*Preparation of Deuterated Holo‐ and Apo‐Az*: We followed our previously published protocol to prepare the deuterated form of holo‐ and apo‐Az.[[qv: 7d]] A solution of holo‐ or apo‐Az, prepared as described above, was diluted (ten times) in a deuterated buffer containing 20 × 10^−3^ m D_3_PO_4_ (D, 99%) and 100 × 10^−3^ m NaOD (D, 99.5%) in D­_2_O (99.9%) at ≈pD7. The diluted solution was repeatedly (ten cycles) concentrated by Amicon Ultracell – 4k (Millipore), rediluted in the deuterated buffer and left overnight. During the ten cycles, the protein was kept in a controlled atmosphere box that was continuously purged with dry N_2_ to maintain 20% relative humidity.

*Cu(I)‐Az Preparation*: Holo‐Az was dialyzed against 50 × 10^−3^ m ammonium acetate buffer, pH 8, containing 100 × 10^−3^ m of ascorbate for ≈6 h. During the process, the blue color of the solution disappeared. Following this the bag was moved to a fresh 50 × 10^−3^ m ammonium acetate buffer, pH 4.6, for an additional 24 h of dialysis. All incubations were conducted in the above‐mentioned controlled atmosphere box to avoid reoxidation by O_2._

**Figure 5:**
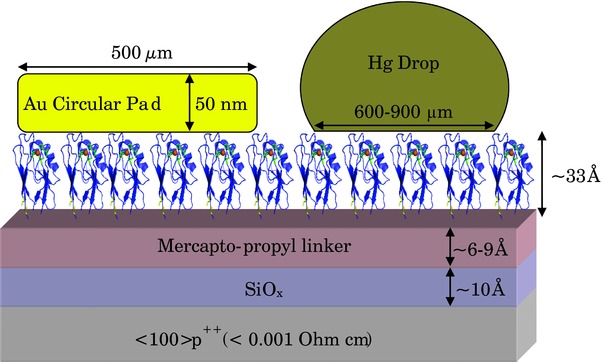
Schematic cross‐section of the experimental setup: the Az molecules are oriented and covalently bound to the substrate via the specific (Cys3 or Cys26) thiol bridges. Because of this all molecules are oriented in the same way with respect to the substrate and the top electrode (although not necessarily in the “soldiers‐on‐a‐parade” manner shown). The highly conducting Si substrate is grounded and voltage is applied to the Au or Hg contact.

*Sample Preparation for ETp Measurements*: The <100> surface of highly doped (<0.001 Ω cm) p‐type Si (P^++^Si) was cleaned by bath sonication by placing the Si surface in a vial containing ethyl acetate/acetone/ethanol (2 min in each), followed by 30 min of piranha treatment (7/3 v/v of H_2_SO_4_/H_2_O_2_) at 80 °C. The Si surface was then thoroughly rinsed in Milli‐Q (18 MΩ) water and dipped in 2% HF solution for 90 s to etch the Si surface (leaving a Si–H surface). For controlled growth of a thin oxide layer (9–10 Å), the etched Si surface was put in a fresh piranha solution for several seconds, and then immediately rinsed thoroughly by Milli‐Q water and dried under a nitrogen stream. A monolayer of 3‐mercaptopropyl trimethoxysilane (3‐MPTMS, SH‐terminated linker) was prepared by immersing the SiO_2_ substrate in 10 × 10^−3^ m 3‐MPTMS in bicyclohexyl solvent for 1 h, followed by 3 min of bath sonication in acetone and 10 s in hot ethanol, yielding a monolayer thickness of ≈7 Å. The latter surfaces were immersed in a vial containing the desired Az variant for ≈6 h. Az was covalently bound to the P^++^ Si/SiOx substrate, via the exposed cysteine residue (Cys3 or Cys26) that was bound to the (≈6 Å) 3‐MPTMS linker molecule (cf. **Figure** **5** for the resulting architecture of the sample). The samples were characterized by varying angle spectroscopic ellipsometry at each immobilization step (thin oxide layer, linker, and protein). Ellipsometry measurements were performed with a Woollam M‐2000V multiple wavelength ellipsometer at an angle of incidence of 70°. A Cauchy model was used (on top of the Si/SiOx/MPTMS layers) to evaluate the protein monolayer thickness. The widths, deduced from the ellipsometry data, were, for all the Az variants, in the range of 19 ± 2 Å, as deduced by ellipsometry.

*Current–Voltage Measurements*: The top contact was made by placing a drop of Hg by capillary on top of the protein monolayer or by depositing 50 nm thick Au pads with lift‐off float‐on (LOFO) technique^51^ (cf. Figure 5). InGa was used as a back contact by scratching the backside of the Si surface and rubbing an InGa eutectic paste onto it. The top contact (Hg or Au) was biased and the back contact was grounded. Thus, both electrical contacts to the protein were electronically conducting and, ionically blocking, i.e., no ionic current is measured during what is essentially a DC experiment. The geometric contact area as measured by an optical microscope was in both cases 0.2 mm^2^, i.e., the same as what was used in the previously reported macroscopic experiments.[[qv: 7e]] ETp is measured by monitoring the current that flows in a two‐terminal configuration, with a monotonically varying bias voltage applied to the top electrode, while the bottom electrode is grounded (Keithley 6430 Sub‐Femtoamp Source‐Meter). Generally current densities that were measured with Hg were lower, up to a factor of 2, than those with Au LOFO (using geometric areas in both cases). For temperature‐controlled measurements, the sample was placed in a low vacuum (≈10^−4^–10^−5^ mbar) chamber in a Model TTPX cryogenic four‐probe electrical measurement system (Lakeshore), and both the sample holder and the probes were cooled, their temperature monitored and controlled ±0.2 K.

## Supporting information

As a service to our authors and readers, this journal provides supporting information supplied by the authors. Such materials are peer reviewed and may be re‐organized for online delivery, but are not copy‐edited or typeset. Technical support issues arising from supporting information (other than missing files) should be addressed to the authors.

Supplementary
