# Supplementary material for: Electron Transfer Proteins as Electronic Conductors: Significance of the Metal and Its Binding Site in the Blue Cu Protein, Azurin
Source: Adv Sci (Weinh). 2015 Mar 16;2(4):1400026. doi: 10.1002/advs.201400026 (PMC5115354; doi:10.1002/advs.201400026)
Supplement: Supplementary file 1 — Supplementary [file ADVS-2-0a-s001.pdf]

Copyright WILEY-VCH Verlag GmbH & Co. KGaA, 69469 Weinheim, Germany, 2015.

ADVANCED SCIENCE
Open Access

Supporting Information

for *Adv. Sci.*, DOI: 10.1002/advs. 201400026

Electron Transfer Proteins as Electronic Conductors: Significance of the Metal and Its Binding Site in the Blue Cu Protein, Azurin

Nadav Amdursky, Lior Sepunaru, Sara Raichlin, Israel Pecht, Mordechai Sheves,* and David Cahen**

Electron Transfer Proteins as Electronic Conductors:

Significance of the metal and its binding site in the blue Cu protein, Azurin

Nadav Amdursky,[†, ‡,a] Lior Sepunaru,[†,a] Sara Raichlin,[†, ‡]

Israel Pecht,[§,*] Mordechai Sheves,[‡,*] David Cahen,[†,*]

Departments of [†]Materials and Interfaces, [‡]Organic Chemistry, and [§]Immunology, Weizmann Institute of Science, Rehovot 76100, Israel

[a]These authors contributed equally.

* Corresponding authors:

Prof. David Cahen

Phone: +972-8-934-2246

e-mail: David.Cahen@weizmann.ac.il

Prof. Mordechai Sheves

Phone: +972-8-934-4320

e-mail: mudi.sheves@weizmann.ac.il

Prof. Israel Pecht

Phone: +972-8-934-4020

e-mail: israel.pecht@weizmann.ac.il

Supplementary Figures

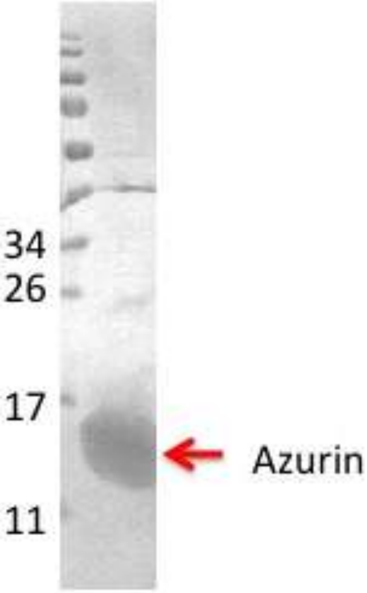

Figure S1. *SDS-electrophoresis gel of the expressed Cu-Az.*

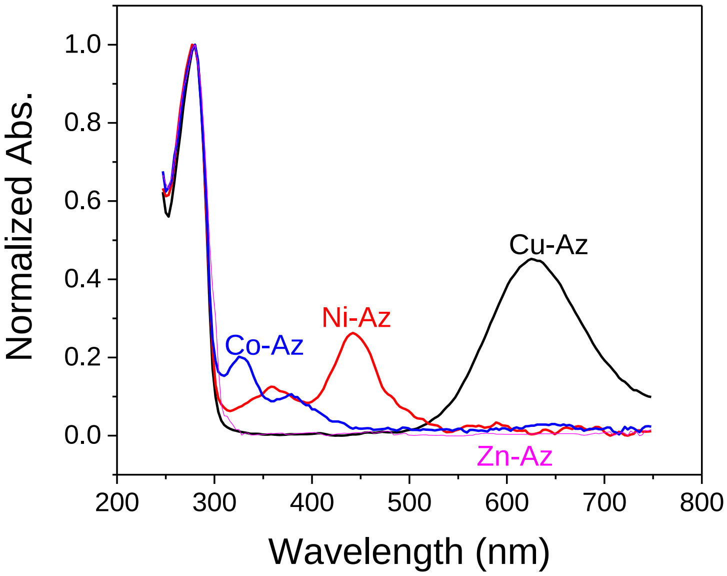

Figure S2. *UV-Vis absorption of Cu-, Ni-, Co and Zn-Az.*

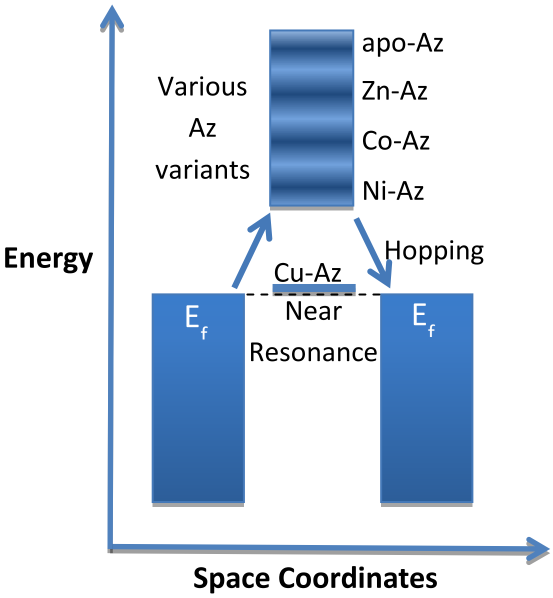

Figure S3. *Simplified energy diagram for the position of the energy levels of the different Az variants (excluding Cu(I) Az), in respect to the electrodes Fermi level, E_f.*

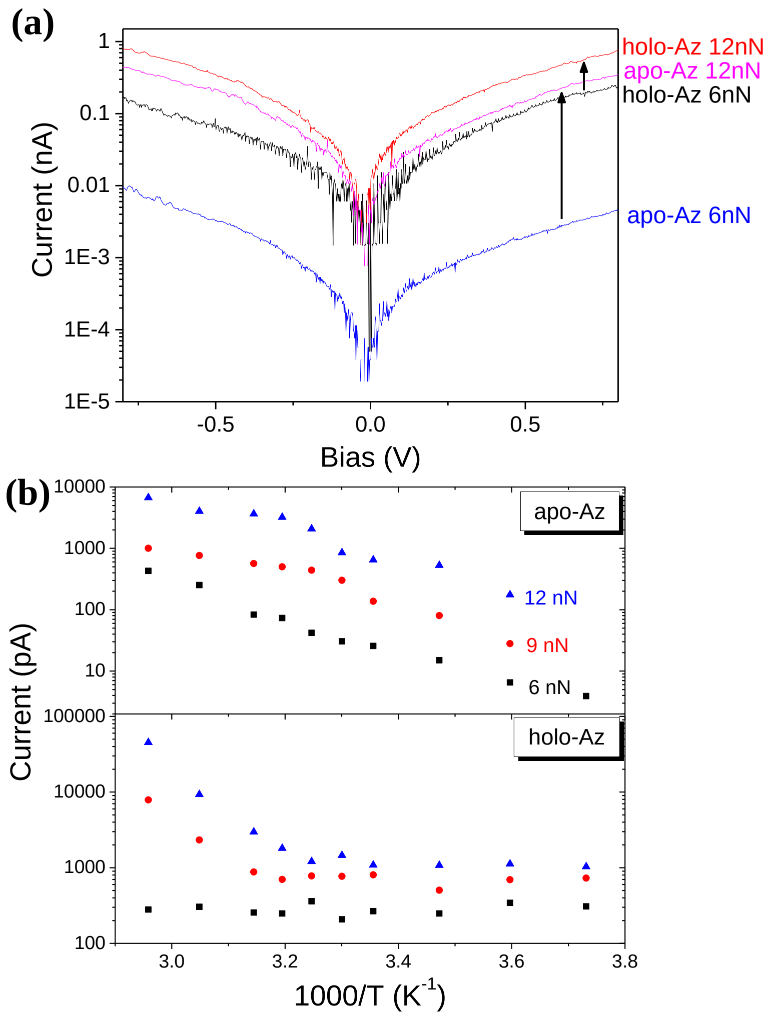

Figure S4. *CP-AFM measurements. (a) I-V of holo-Az and apo-Az at applied tip forces of 6 and 12 nN. (b) ln(I) vs. 1000/T plots (at applied bias of 0.5V) curves of holo-Az and apo-Az at 6, 9 and 12 nN applied tip forces. Reconstructed from Ref.*[7h]
